# Antineoplastic Activity of *Methyl rosmarinate* in Glioblastoma Cells

**DOI:** 10.3390/cimb47030180

**Published:** 2025-03-10

**Authors:** Maria Vasiliki Benekou, Panagiota Tzitiridou, Theodora Papagrigoriou, Vasiliki Galani, Chrissa Sioka, Athanassios P. Kyritsis, Diamanto Lazari, George A. Alexiou

**Affiliations:** 1 Neurosurgical Institute, University of Ioannina, 45500 Ioannina, Greece; bl02304@uoi.gr (M.V.B.); tzitiridoupanagiota@gmail.com (P.T.); csioka@uoi.gr (C.S.); thkyrits@uoi.gr (A.P.K.); 2 Laboratory of Pharmacognosy, School of Pharmacy, Faculty of Health Sciences, Aristotle University of Thessaloniki, 54124 Thessaloniki, Greece; tpapagri@pharm.auth.gr (T.P.); dlazari@pharm.auth.gr (D.L.); 3 Department of Anatomy Histology-Embryology, School of Medicine, University of Ioannina, 45110 Ioannina, Greece; vgalani@uoi.gr; 4 Department of Neurosurgery, University of Ioannina, 45500 Ioannina, Greece

**Keywords:** glioblastoma, *Methyl rosmarinate*, organic compounds, cancer, glioma, *Thymus thracicus*

## Abstract

Glioblastoma (GMB) is a remarkably aggressive brain malignancy characterized by high mortality rates, despite continuous advances in therapeutic approaches. Compounds derived from plants are being studied for their potent medicinal properties in the quest for more efficient therapies. This study investigated the anti-glioma properties of *Methyl rosmarinate*, a hydroxycinnamic acid isolated from *Thymus thracicus* Velen, which has previously demonstrated anti-cancer activity in various cell lines. Human glioblastoma cell lines U87 and T98 were treated with *Methyl rosmarinate* to assess its effect on cell viability, cell cycle distribution and migratory capacity using Trypan blue assay, flow cytometry and scratch wound healing assay, respectively. The combinatorial effects of *Methyl rosmarinate* and temozolomide were also analyzed with CompoSyn software. According to the outcomes, *Methyl rosmarinate* significantly reduced cell viability, induced cell death by interfering in cell cycle checkpoints, and inhibited migration in both GMB cell lines. Notably, in U87 cells, the compound showed a synergistic impact with temozolomide, whereas in T98 cells, there was an antagonistic relationship. These results suggest that *Methyl rosmarinate* has potential anti-glioma properties; however, more in vivo research is needed.

## 1. Introduction

Glioblastoma (GMB) is the most prevalent malignant primary brain tumor, resulting in thousands of deaths globally each year. GBM is a WHO grade 4 brain tumor that is thought to originate from mutations in neural stem cells [1] and accounts for 15 to 20% of all primary intracranial neoplasms in adults [2]. Despite the availability of modern treatments, including surgical resection combined with radiotherapy and chemotherapy, the average survival time for patients with GBM is still less than 20 months [3]. Both the biological characteristics of GBM (inter-tumor and intra-tumor heterogeneity, heterogeneous permeability of the blood–brain barrier and immunosuppressive tumor microenvironment) as well as the growing resistance to commercially available chemotherapeutics, especially temozolomide (TMZ), the first-line treatment for GBM, make medical therapy challenging [4,5,6]. The persistent difficulties in treating glioblastoma underline the need for novel, more effective treatments. Studies on plant-based and organic compounds are becoming increasingly popular when standard treatments have proven inadequate.

Since ancient times plants have always been the basis for traditional medicine systems and have provided continuous cures for mankind for thousands of years. Medicinal plants offer various bioactive compounds (alkaloids, terpenoids, flavonoids, phenolic compounds, etc.) that have been used for thousands of years because of their therapeutic properties [7]. Herbal products have been utilized in healthcare for years, especially since several plant compounds have demonstrated potential activity against various cancers. Anticancer agents of plant origin that are already in clinical use around the world include vincristine, taxol, vinblastine and camptothecin [8]. Natural compounds have already attracted researchers’ attention as it comes to glioblastoma, and related research showed that a variety of plant-isolated agents inhibit cell growth and cause apoptosis in glioblastoma cells [9].

**Figure 1:**
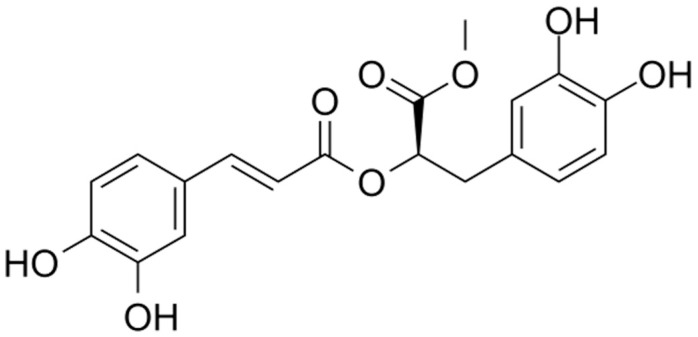
Structure of *Methyl rosmarinate*.

*Methyl rosmarinate* (RM) was isolated from the plant *Thymus thracicus* Velen. *Thymus thracicus* is an evergreen perennial herb with lavender flowers in summer. It is a Mediterranean plant that mainly grows in Greece (North Aegean islands, North Central Greece, North Pindos, North-East Greece, South Pindos, and the West Aegean islands). It can reach up to 6 to 12 cm in height and 15 to 18 cm in width. It is resistant to deer and rabbits, as well as attracting bees and butterflies. To thrive, it needs sun and frequent to sporadic watering. In the summer, it prefers to be dry. It grows in open rocky slopes at elevations between 500 and 1800 m, as well as in meadows with coarse, well-drained soils [10,11]. RM belongs to the hydroxycinnamic acids, a class of aromatic acids or phenylpropanoids with a C6-C3 skeleton—hydroxy derivatives of cinnamic acid (Figure 1). RM, as an ester derivative of the well-studied Rosmarinic Acid [12], appears to share many of its properties, exhibiting a variety of antioxidant, anti-inflammatory, antimicrobial, anticancer and neuroprotective properties [13,14,15,16].

*Methyl rosmarinate* is beneficial for a variety of diseases such as diabetes, acting as a glucosidase inhibitor [17] and for asthma and inflammatory bowel disease due to its lipoxygenase inhibitory action [18]. In recent research, it has been associated with the treatment of SARS-CoV-2 virus as it acts as an allosteric inhibitor of the 3-chymotrypsin-like protease (3CLpro) that plays a critical role in viral replication, showing its antivirus potential [19]. RM, additionally, appears to have therapeutic activity in various neurodegenerative diseases as it acts as an inhibitor of cholinesterase, an enzyme that hydrolyses acetylcholine, a neurotransmitter involved in cognitive function [20].

Regarding cancer, a plethora of research has demonstrated RM as a potential therapeutic anti-cancer agent. RM inhibits the growth of leukemic cells (HL-60, Jurkat, K562 and CCRF-SB), breast cancer cells (MCF-7, MDA-MB-231) [16] and human hepatoma cell lines (Hep-3B and SK-Hep1) [21]. In the current study, we investigated the RM anti-cancer effects in glioblastoma cell lines in combination with temozolomide.

## 2. Materials and Methods

### 2.1. Isolation and Identification of Methyl rosmarinate

**Figure 2:**
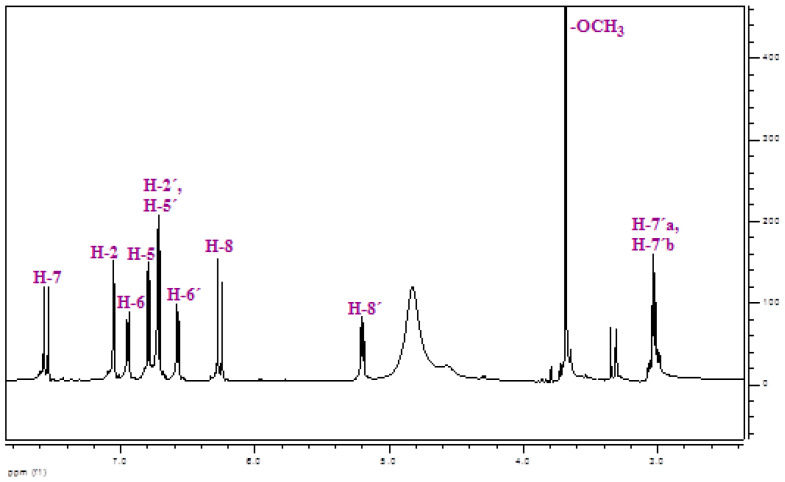
^1^H-NMR spectrum of *Methyl rosmarinate* (CD_3_OD, 500 MHz).

*Methyl rosmarinate* was isolated from the ethyl acetate fraction of methanol extract of *Thymus thracicus* Velen (Lamiaceae), which was collected from the wild from Mt. Paggaio in Northwest Greece and in continuation submitted to extraction according to Tzitiridou et al. 2024 [22]. Fraction D of the ethyl acetate fraction (1487.5 mg) was subjected to silica column chromatography Silica 9385 (Merck Art. 9385, Darmstadt, Germany) and eluted with mixtures of Dichloromethane (DM)/Methanol (MeOH)/Water (H_2_O), yielding 13 fractions (DA-DM) from which the fraction DE eluted with DM:MeOH:H_2_O 90:10:1 (198.3 mg) was identified as *Methyl rosmarinate* by means of NMR spectroscopy. Thin Liquid Chromatography (TLC) was used to control the quality of the fractions. For the TLC, a silica gel (Kieselgel F254, Merck, Art. 5554) stationary phase on aluminum foil (20 cm × 20 cm, 0.1 mm) with a fluorescence marker was used. The development of the TLC plates was carried out using mixtures of solvents appropriate for each group of fractions. Finally, the TLC plates were sprayed with vanillin-H_2_SO_4_ (1:1). The identification/verification of *Methyl Rosmarimate* was performed via ^1^H-NMR studies. The NMR spectra were recorded in CD_3_OD using an AGILENT DD2 500 (500.1 MHz for ^1^H-NMR) spectrometer. Chemical shifts are reported in δ (ppm) values relative to TMS (3.31 ppm for ^1^H-NMR for CD_3_OD). The data of isolated and identified *Methyl rosmarinate* (Table 1 and Figure 2) were compared with those of samples from our collection and/or with reported data in the literature [23].

### 2.2. Cell Lines and Conditions for Treatment

The U87 and T98 human glioma cell lines were acquired by Dr. W.K. Alfred Yung from the Department of Neuro-Oncology at M.D. Anderson Cancer Center in Houston, TX, USA, and ATCC from Manassas, VA, USA, respectively. Supplemented Dulbecco’s Modified Eagle’s Medium (Gibco BRL, Life Technologies, Grand Island, NY, USA) with 10% fetal bovine serum (FBS) and 1% penicillin streptomycin (Gibco BRL) was used for the cultivation of both cell lines. Cells were incubated under humidified conditions with 5% CO_2_ at 37 °C. *Methyl rosmarinate* was isolated from the *Thymus thracicus*’ methanolic extract, diluted with DMSO, and stored at −80 °C. TMZ obtained from Sigma Aldrich was diluted in DMSO and stored at 20 °C. DMEM was used to dilute RM and TMZ from stock solutions to the final concentration before each experiment.

### 2.3. Cell Viability Assay

The Trypan Blue assay was utilized to evaluate the cell viability of RM in both the U87 and T98 cell lines, as described in our previous studies [24]. About 20,000 cells were seeded in 12-well plates, and after 24 h of incubation, they were treated with RM in concentrations of 5, 15, 30, 45 and 60 μΜ. Trypan blue stain was added after 72 h and then phase contrast microscopy was used to assess cell viability. Furthermore, a Trypan Blue exclusion assay was used to evaluate the viability of the cells following combination therapy with TMZ.

### 2.4. Flow Cytometric Analysis of DNA Cell Cycle

About 20,000 cells were seeded for the DNA cell cycle analysis. After 24 h, the U87 cells were treated with 9.8 μM and 19.6 μM RM and T98 cells with 13 μM and 26 μM RM for an additional 72 h. Afterwards, cells were rinsed twice with PBS solution, treated with trypsin and incubated with PI (Propidium Iodide) working solution (50 g/mL PI, 20 mg/mL RNaseA, and 0.1% Triton X-100) at 37 C for 20 min. A flow cytometer (Omnicyt, Cytognos S.L., Grand Island, NY, USA) was used to gather the PI fluorescence data of the cell’s nuclei and then cell cycle phases were analyzed.

### 2.5. Combination Treatment with RM and TMZ

In order to assess whether RM and TMZ synergize, T98 and U87 cells were treated with various combinations of these two compounds. The cells were cultivated on 24-well plates, and after 24 h they were exposed to four different concentrations of RM and TMZ and their combination, ranging from half to twice IC50. The U87 cells were treated with 4.9, 9.8, 14.7, 19.6 μM RM and 25, 50, 75, 100 μM TMZ and T98 cells with 6.5, 13, 19.5, 26 μM RM and 60, 120, 180, 240 μM TMZ. The concentrations of the chemicals added up progressively from lowest to highest. Viability was determined using the trypan blue exclusion assay after 72 h. Three repetitions of each condition were tested for both series. The Chou–Talalay combination index approach was used to assess the combined effect of TMZ and RM. Following treatment with RM alone, TMZ alone, or various combinations of the two, the affected fraction (Fa) of cells was determined, and dose–effect curves were generated. The software CompuSyn was used to determine the Combination Index (CI). The CI value illustrates the effect of the combined treatment. It has been established that the effect is antagonistic for CI > 1, additive for CI = 1, and synergistic for CI < 1 [25].

### 2.6. Scratch Wound Healing Assay

10^5^ U87 and T98 cells were seeded in each well of a 6-well plate, and the cells were incubated for 24 h in a medium containing 10% FBS. The cells were then allowed to starve for a further 24 h in a medium containing 1% FBS. Following two PBS washes, each well was filled with fresh media containing 1% FBS. A 200-L sterile pipette tip was used to create the scratch wound by scraping each well from top to bottom. Next, IC50 and twice IC50 concentrations of RM were applied. Phase contrast microscopy was used at 0, 24 and 48 h to capture pictures of the cells that moved into the region of the scratch wound that was free of cells.

### 2.7. Statistical Analysis

The data were expressed using the mean and standard deviation. We utilized non-linear regression analysis with GraphPad Prism software (v. 8.0.0, San Diego, CA, USA, Trial Version) to calculate the IC50 value of RM. Microsoft Excel was used to do the statistical analysis. When *p* < 0.05, differences were deemed significant. We measured the spacing between the scratch edges using ImageJ software (version 8.0).

## 3. Results

### 3.1. Calculation of Half Maximal Inhibitory Concentration of Methyl rosmarinate and GMB Viability

**Figure 3:**
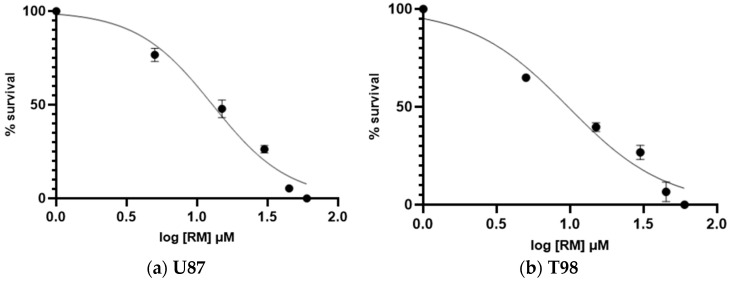
The viability of glioblastoma cells following treatment with the compound *Methyl rosmarinate* (**a**) U87 cells, (**b**) T98 cells. The y axis represents the percentage of cells that survived after treatment with RM and the x axis the logarithm of RM concentration.

**Figure 4:**
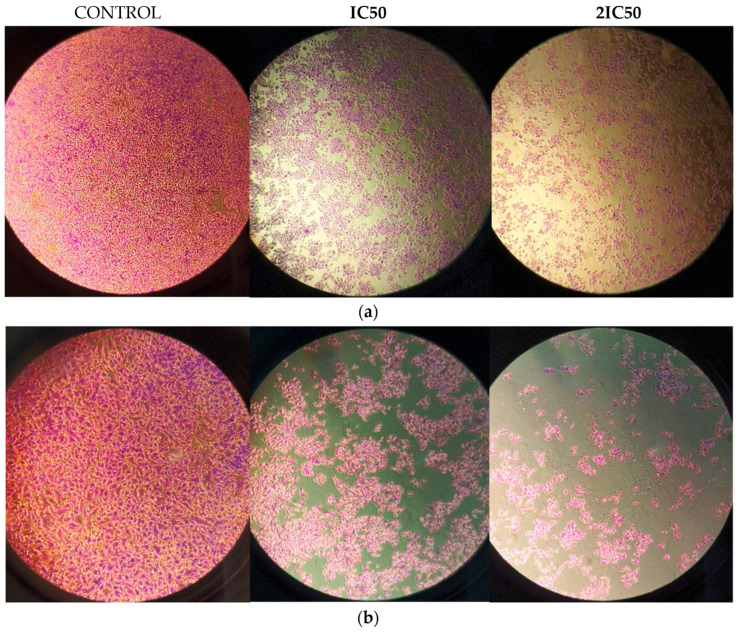
Morphology of U87 cells after treatment with RM concentration equal to IC50 = 9.8 μΜ and 2IC50 = 19.6 μΜ with 0.2% Crystal Violet staining. 10^5^ cells were seeded in 6 well plates. After 24 h RM doses were added following by Crystal Violet staining in 72 h. Magnification 5× (**a**) and 10× (**b**).

**Figure 5:**
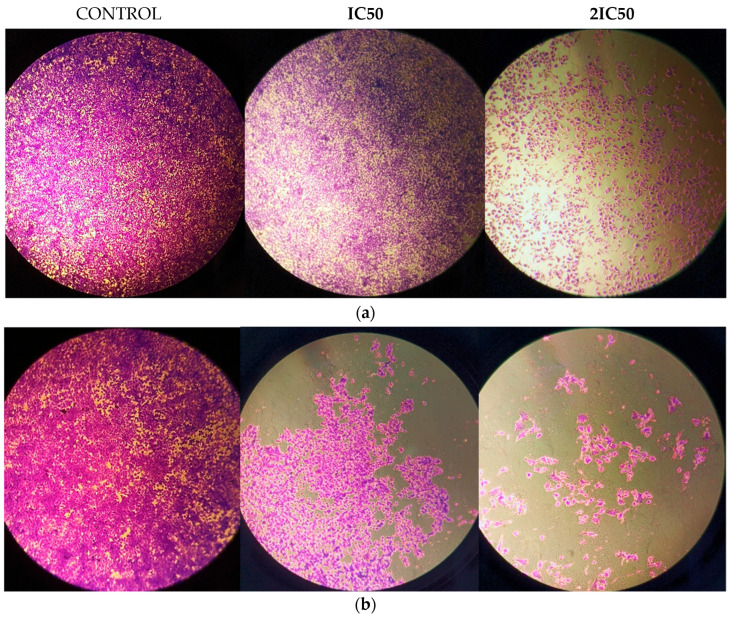
Morphology of T98 cells after treatment with RM concentration equal to IC50 = 13 μΜ and 2IC50 = 26 μΜ with 0.2% Crystal Violet staining. 10^5^ cells were seeded in 6 well plates. After 24 h RM doses were added following by Crystal Violet staining in 72 h Magnification 5× (**a**) and 10× (**b**).

The anti-cancer activity of RM against glioblastoma was assessed by incubating U87 and T98 cells with increasing doses of the agent (5, 15, 30, 45 and 60 μΜ) for a total of 72 h. Cells were counted at all concentrations using the Trypan Blue exclusion test, which distinguishes between live and dead cells, as well as a hemocytometer. The percentage of viability and the concentration that causes 50% of cell death, or IC50, were then calculated. The IC50 value of RM for decreased viability in T98 cells and U87 cells was 13 μM and 9.8 μM, respectively (Figure 3). Under a microscope, cells displayed changes such as shrinking and death when RM concentrations were raised (Figure 4 and Figure 5).

### 3.2. S and G2/M Cell Cycle Arrest Caused by Methyl rosmarinate

**Figure 6:**
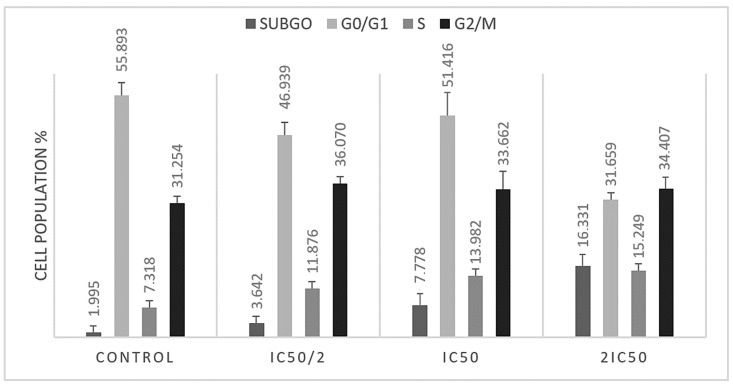
Graphical representation of the percentage of U87 cells in each cell cycle phase after IC50 and twice the IC50 RM dose.

**Figure 7:**
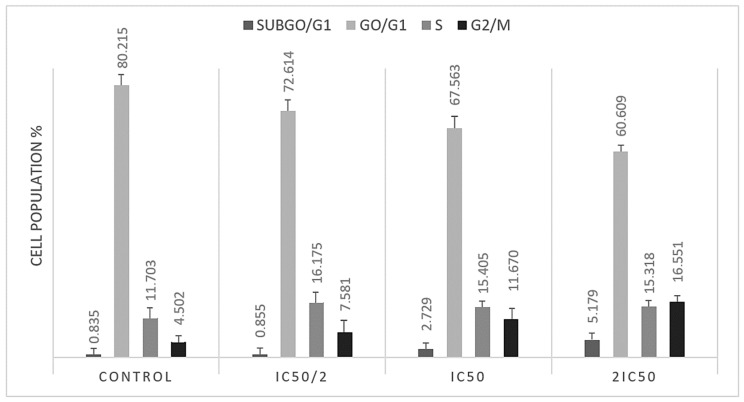
Graphical representation of the percentage of T98 cells in each cell cycle phase after IC50 and twice the IC50 RM doses.

In order to assess the impact of *Methyl rosmarinate* on cell cycle progression in T98 and U87 glioblastoma cells, we performed flow cytometry analysis. Both cell lines were treated with IC50 and twice IC50 values of RM for 48 h. The analysis results indicated that RM induced accumulation in subG0 and S cell cycle phases in U87 (Figure 6) and in subG0 and G2/M in T98 (Figure 7) cells. Particularly, U87 cells showed increased percentage distribution from 1.9945% ± 0.448 to 16.331% ± 1.287 in subG0/G1 phase and from 7.318% ± 0.316 to 15.249% ± 0.544 in S phase (Table 2). T98 cells increased from 0.835% ± 0.212 to 5.179% ± 0.441 in subG0/G1 phase and from 4.502 ± 0.256 to 16.551 ± 0.315 in G2/M phase (Table 3).

### 3.3. Methyl rosmarinate Inhibited U87 and T98 Cell Migration

**Figure 8:**
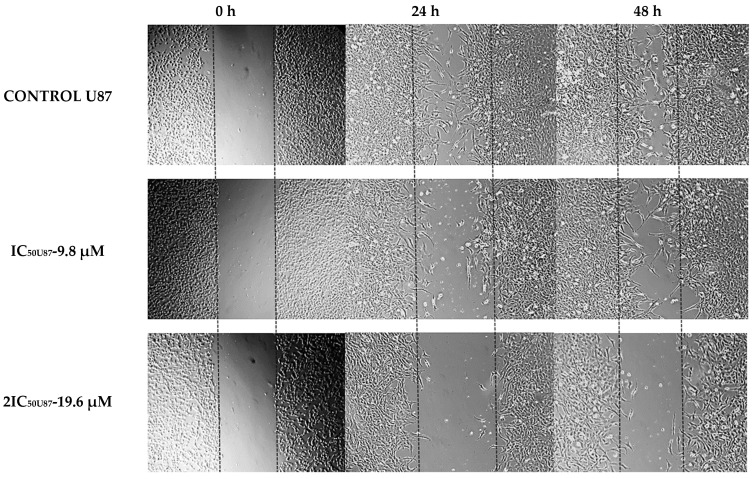

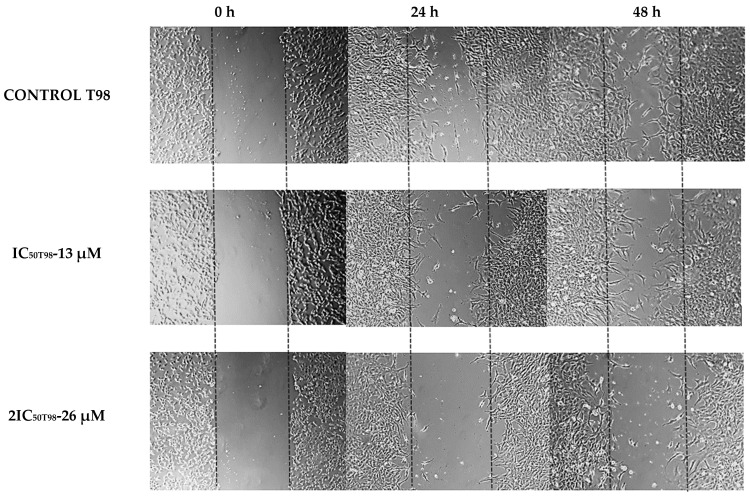
The impact of RM in U87 and T98 cells’ ability to migrate at 0, 24 and 48 h after treatment with IC50 and twice IC5O value. The parallel lines have been set in accordance with the 2IC5O opening. Magnification 5×.

**Figure 9:**
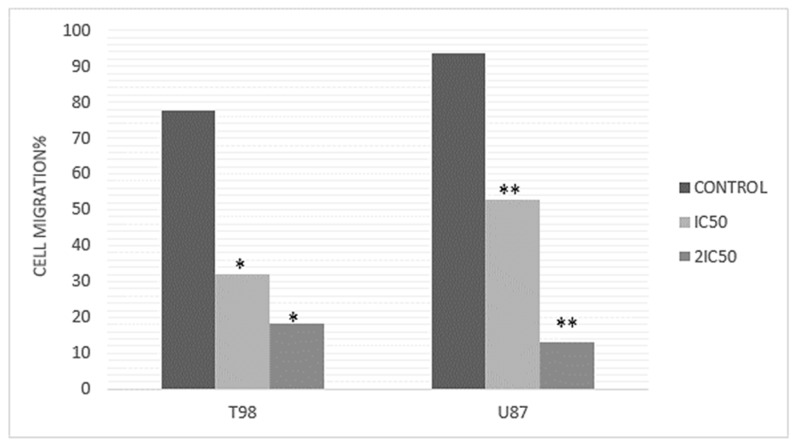
Rates of the ability of the GMB cells to heal the scratched area at 48 h. The wound widths were set to 0% at 0 h and the percentage of migration was estimated by the formula: Width migration = Width0 h–Width48 h. Three repetitions of the experiment were conducted. * *p* < 0.05, ** *p* < 0.01.

Since metastatic progression is the primary cause of death for cancer patients, the study of cell migration in cancer research is highly important. In this case, scratch wound healing assay experiments were conducted with the agent at IC50 and 2×IC50 doses, with photographs taken at 0, 24 and 48 h. The cell migration rate of T98 and U87 cells with increasing RM concentrations was measured based on the narrowing of the gap on the GBM cells monolayer. The results indicated that cells without the agent exhibited great motility and covered the scratch area, whereas incubation with the compound hindered cell motility and reduced coverage (Figure 8). In particular, following exposure to 9.8 μM and 19.6 μM of *Methyl rosmarinate* in U87 cells, the percentage of closure of the wound was 52.8% and 13.2% after 48 h of incubation, respectively, while the while comparing the percentage of the control group was 93.5%. As it comes to the T98 cell line, the results showed 32.12% and 18.3% wound healing closure after treatment with 13 μM and 26 μM *Methyl rosmarinate* whereas the control group managed to heal 77.65% of the scratched area (Figure 9).

### 3.4. Methyl rosmarinate and TMZ Have a Synergistic Effect on the U87 Cell Line

**Figure 10:**
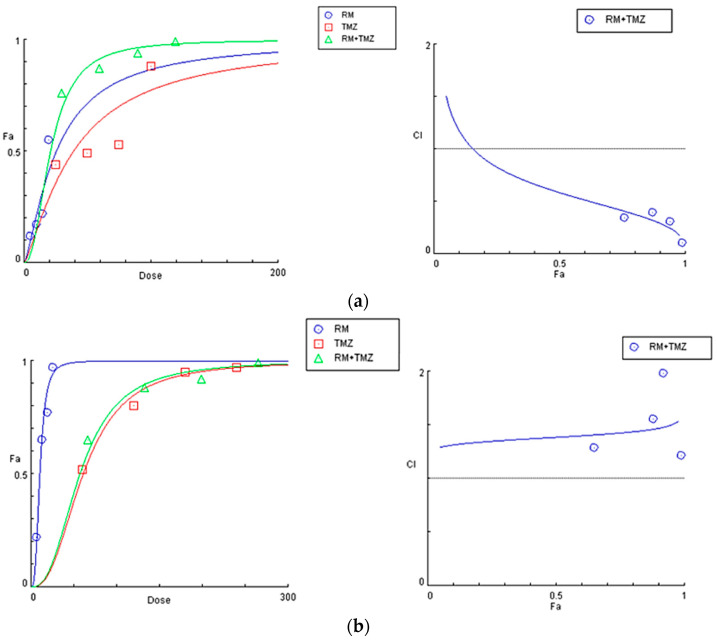
CompuSyn Report’s graphic depiction of the combined effect of the RM and TMZ in U87 (**a**) and T98 (**b**) cells. The dose–effect curve for each compound and the combination of them. The concentrations of compounds are shown on the x-axis, while the effect (% mortality) is shown on the y-axis (left). A plot of the combination index (CI) for each RM + TMZ combination (right).

In order to assess the synergism or antagonism of RM in combination with the first line drug in glioblastoma treatment, temozolomide, both U87 and T98 cells were treated with increasing doses of these compounds (0.5 IC50, IC50, 1.5 IC50 and 2 IC50). U87 cells were treated with 4.9, 9.8, 14.7 and 19.6 μM RM and 25, 50, 75 and 100 μM TMZ and T98 cells with 6.5, 13, 19.5 and 26 μM RM and 60, 120, 180 and 240 μM TMZ. Results were analyzed using the median-effect equation in CompuSyn software. CompuSyn software was used to create dose–effect curves and combination-index plots that graphically depict the combined effect of *Methyl rosmarinate* and TMZ (Figure 10). Outcomes displayed that RM had both synergistic and antagonistic effects. *Methyl rosmarinate* indicated a synergistic effect in all tested concentrations on U87 cells and antagonism in all combinations on T98 cells (Table 4). CompuSyn calculates the dose reduction index for each combination of substances. An additive effect is defined by CI = 1, synergism by CI < 1, and antagonism by CI > 1.

## 4. Discussion

In adults, GMB is the most aggressive primary brain tumor. Despite the standard treatment protocol, which includes maximal surgical resection, temozolomide (TMZ) chemotherapy and radiotherapy, GBM has a poor prognosis and a mean survival period of only approximately 15 months. The tumor’s adaptive abilities and resistance to therapy are mostly caused by the blood–brain barrier (BBB), the extremely diverse nature of GBM and the complex tumor microenvironment. These factors collectively account for the poor efficacy of these conventional treatments [26]. Therefore, novel therapy strategies that can successfully address these issues and enhance patient outcomes are needed.

In recent years, organic compounds obtained from plants have emerged as a promising field of study for the development of innovative anti-glioma treatments. These compounds have demonstrated the ability to protect glial cells by lowering oxidative stress and neuroinflammation, and they are typically less hazardous than synthetic medicines [27]. Furthermore, it has been shown that substances derived from plants have the ability to hinder crucial oncogenic processes in GBM, like cell survival and proliferation, by triggering apoptotic pathways and cell cycle arrest [28].

Notably, it has been discovered that a few of these natural medicines, especially those with molecular weights below 450–500 Da, can penetrate the blood–brain barrier, which makes them especially useful in exerting their potential neuroprotective properties and contribute to the treatment of brain cancers [29]. Furthermore, through their synergistic actions, they can increase the efficacy of conventional treatments like radiation and TMZ, which offers a viable way to overcome GBM’s inherent resistance and prolong patient lives. Therefore, further research into herbal remedies holds significant potential for developing more effective multimodal treatment approaches for glioblastoma.

RM is a hydroxycinnamic acid, a class of aromatic acids or phenylpropanoids that is demonstrating a wide range of anti-inflammatory, antibacterial, neuroprotective, antioxidant, antivirus and anticancer effects. It has known antimicrobial and bactericidal activity mainly on Gram positive bacteria, as it interferes with microbial cell membranes by disrupting bacterial growth [14,15,16]. It also exhibits antioxidant and anti-inflammatory properties, protecting cells from oxidative stress by removing free radicals and reducing inflammation by inhibiting pro-inflammatory cytokines [17].

In the context of molecular mechanisms, RM exhibits an inhibitory effect on metalloprotease 1, an enzyme critical in cancer progression [30]. Furthermore, the compound interacts with molecular targets and pathways involved in hepatocellular carcinoma HCC, including PIK3R1 (key player in the PI3K pathway), RELA (subunit of NF-κB), EGFR (epidermal growth factor receptor) and EP300 (histone acetyltransferase), all of which are essential for cellular functions like survival, proliferation and apoptosis [31,32].

Research findings involving liver cancer cells (Hep-3B and SK-Hep1) and healthy liver cells (MIHA) treated with RM showed that RM induced apoptosis, as evidenced by increased expression of pro-apoptotic proteins (Bax and cleaved PARP) and decreased expression of the anti-apoptotic protein Bcl-2. Furthermore, RM treatment was found to inhibit the migration and invasion of liver cancer cells and led to alterations of epithelial-mesenchymal transition (EMT) markers. Importantly, it was revealed that RM reduced the phosphorylation of Akt and mTOR proteins, suggesting inhibition of the Akt/mTOR signaling pathway [23].

In addition, *Methyl rosmarinate* has been shown to interact with proteins involved in intracellular signaling pathways, such as MEK, which is crucial for cell proliferation, differentiation and survival, and it has been found to target proteins involved in angiogenesis, such as VEGFR2. Emphasizing its potential anticancer effects, inhibition of VEGFR2 signaling can inhibit the formation of new blood vessels, which is essential for tumor growth and metastasis [33]. Collectively, the findings from the literature indicate that *Methyl rosmarinate* affects several pathways that are often dysregulated in cancer, including EGFR/PI3K/AKT/mTOR and MAPK/ERK, as well as the angiogenesis process. [34,35,36].

In the present study, we evaluated the cytotoxic effects of *Methyl rosmarinate*, a hydroxycinnamic acid isolated from thymus, on GBM cell lines. Our results show that RM, with relatively low IC50 values in both tested cell lines, significantly and dose-dependently decreased cell viability and proliferation. Furthermore, RM caused cell cycle arrest in both cell lines and an increase of the subG0/G1 indicating apoptosis.

Our investigation also demonstrated that RM significantly reduced cell migration. One of the main reasons why GBM therapies are inefficient is because of cancer cells’ migratory ability. Cancer cells can migrate even after a successful surgical procedure. RM significantly decreased the T98 and U87 cell lines’ ability to migrate in a manner that was both dose- and time-dependent as compared to the controls [36].

Regarding the combined treatment, RM and TMZ had both synergistic and antagonistic relationships in the U87 and T98 cell lines, respectively. Generally, antagonism is more likely to occur when two drugs saturate the effect that the other medication would typically have, target the same phase of the cell cycle via similar mechanisms, or act in opposition to one another on the same targets [37]. *Methyl rosmarinate*’s different effects when combined with TMZ in U87 and T98 cells can also be related to the distinct molecular characteristics of the two cell lines, such as MGMT expression and *p53* status [38,39].

Further experiments are necessary to have a deeper understanding of these interactions. Western blot investigations may shed light on changes in protein expression linked to pathways for apoptosis and cell cycle control. Determining the methylation status of the MGMT promoter in T98 cells may help explain TMZ resistance mechanisms [40]. Moreover, gene expression profiling may reveal differentially affected RM genes and pathways in U87 and T98 cells. Assays for drug efflux and live-cell imaging may provide real-time information on drug dynamics and metabolism [41]. These studies will be crucial for enhancing combination treatments and comprehending the fundamental mechanisms influencing medication efficacy in glioblastoma treatment.

## 5. Conclusions

This research indicates that *Methyl rosmarinate* has notable cytotoxic and anti-migratory effects on glioma cell lines, suggesting a possible treatment for glioblastoma. Nonetheless, the varying interactions between RM and TMZ, specifically the antagonistic effect found in T98 cells, underscores the intricacy of combination therapies and the necessity for extended assessment. To optimize treatment approaches, more investigation is needed into the molecular mechanisms behind these effects, including DNA repair, cell cycle dynamics, and drug resistance pathways. Comprehending these interactions will be critical to optimizing drug combinations to improve the effectiveness of glioblastoma therapies and consequently patients’ quality of life.

## Figures and Tables

**Table 1:** ^1^H-NMR of *Methyl rosmarinate* (CD_3_OD, 500 MHz).

δ (ppm)	Multiplicity	J (Hz)	n^o^ H	Identification
7.55	*d*	16.1	1	H-7
7.05	*d*	2.0	1	H-2
6.94	*dd*	8.3, 2.0	1	H-6
6.79	*d*	8.3	1	H-5
6.72	*d*	2.0	1	H-2′
6.71	*d*	8.3	1	H-5′
6.57	*dd*	8.3, 2.0	1	H-6′
6.26	*d*	16.1	1	H-8
5.20	*dd*	7.4, 4.8	1	H-8′
3.69	*s*	-	3	-OCH_3_
3.05	*dd*	14.1, 4.8	1	H-7′a
3.01	*dd*	14.1, 7.4	1	H-7′b

**Table 2:** Results of cell cycle analysis by flow cytometry technique in U87 after treatment with RM. Quantification of cells in different cell cycles is shown expressed as mean ± SD from three different experiments. * *p* < 0.05.

%	subG0	G0/G1	S	G2/M
Control	1.9945 ± 0.448	55.893 ± 1.975	7.318 ± 0.316	31.254 ± 1.079
IC50/2	3.642 ± 0.523 *	46.939 ± 2.420 *	11.876 ± 0.732 *	36.070 ± 0.653 *
IC50	7.778 ± 1.676 *	51.083 ± 6.369 *	13.982 ± 1.019 *	35.181 ± 3.801 *
2IC50	16.331 ± 1.287 *	31.659 ± 1.084 *	15.249 ± 0.544 *	34.407 ± 0.742 *

**Table 3:** Results of cell cycle analysis by flow cytometry technique in T98 cells after treatment with RM. Quantification of cells in different cell cycles is shown, expressed as mean ± SD from three different experiments. * *p* < 0.05.

%	subG0	G0/G1	S	G2/M
Control	0.835 ± 0.212	80.215 ± 1.549	11.703 ± 1.188	4.502 ± 0.256
IC50/2	0.855 ± 0.076 *	72.614 ± 2.291 *	16.175 ± 1.016 *	7.581 ± 1.735 *
IC50	2.729 ± 0.906 *	67.563 ± 2.263 *	15.405 ± 0.686 *	11.670 ± 1.264 *
2IC50	5.179 ± 0.441 *	60.609 ± 0.149 *	15.318 ± 0.304 *	16.551 ± 0.315 *

**Table 4:** Evaluation of the combinatorial effect of RM and TMZ in U87 and T98 cells. Data were generated using CompuSyn software. The data show the concentrations of each substance, the effect (%mortality), and the CI of each combination. CI permits quantitative determination of drug interactions, where CI < 1, =1, and >1 indicate synergism, additive effects and antagonism, respectively.

*Methyl rosmarinate* RM (μΜ)	Temozolomide ΤΜΖ (μΜ)	Effect	CI	Conclusion
**U87 cells**				
4.9	25	0.76	0.34767	SYNERGY
9.8	50	0.87	0.39376	SYNERGY
14.7	75	0.94	0.3095	SYNERGY
19.6	100	0.99	0.10174	SYNERGY
**T98 cells**				
6.5	60	0.65	1.28268	ANTAGONISM
13	120	0.88	1.5547	ANTAGONISM
19.5	180	0.92	1.98022	ANTAGONISM
26	240	0.99	1.21169	ANTAGONISM

## Data Availability

The author confirms that all data generated or analyzed during this study are included in this published article.

## Abbreviations

CD_3_OD	Deuterated Methanol
CI	Combination Index
BBB	Blood–brain barrier
DMSO	DimethylSulfoxide
FBS	Fetal Bovine Serum
GBM	Glioblastoma
H_2_SO_4_	Sulfuric acid
MEK	Mitogen-activated protein kinase
NMR	Nuclear Magnetic Resonance
PBS	Phosphate-Buffered Saline
PI	Propidium Iodide
PI3K	Phosphoinositide 3—kinase
RM	*Methyl rosmarinate*
TMZ	Temozolomide
VEGFR2	Vascular Endothelial Growth Factor Receptor 2
